# The inflammatory state provokes sexual dimorphism in left ventricular and electrocardiographic effects of chronic cyclosporine in rats

**DOI:** 10.1038/srep42457

**Published:** 2017-02-13

**Authors:** Hany M. El-Bassossy, Zainy M. Banjar, Mahmoud M. El-Mas

**Affiliations:** 1 Department of Pharmacology, Faculty of Pharmacy, King Abdulaziz University, Jeddah, Kingdom of Saudi Arabia; 2 Department of Pharmacology, Faculty of Pharmacy, Zagazig University, Egypt; 3 Departement of Clinical Biochemistry, Faculty of Medicine, King Abdulaziz University, Jeddah, Kingdom of Saudi Arabia; 4 Department of Pharmacology and Toxicology, Faculty of Pharmacy, Alexandria University, Egypt

## Abstract

Although cardiotoxicity has been recognized as an adverse effect of cyclosporine A (CSA), no information exists regarding sex specificity of CSA cardiotoxicity. We tested the hypothesis that left ventricular (LV) and electrocardiographic (ECG) effects of CSA and related inflammatory/histopathological derangements are sex related. CSA reduced the LV slope of end-systolic pressure volume relationship and increased isovolumic relaxation constant. These effects were more pronounced in male compared to female rats, suggesting LV systolic and diastolic dysfunction. ECG recordings showed elevated ST segments and increased QTc and T peak trend intervals in CSA-treated male rats, markers of LV ischemia and arrhythmogenesis. In female rats, CSA delayed AV conduction, as reflected by prolonged PR interval. Other sex-related effects for CSA included (i) increased blood cholesterol, and reduced rates of rise and fall in LV pressure and nuclear factor kappa B and angiotensin receptors type 1 expressions in male rats, and (ii) increased LV adiponectin in females. Histopatholgically, CSA caused vascular congestion, blood extravasation, and pyknotic or even absent nuclei in both sexes. In conclusion, rats exhibit sex-independent susceptibility to negative LV and histopathological influences of CSA. These effects become more intensified in male rats, perhaps on account of aggravated ischemic and inflammatory milieus.

Cyclosporine A (CSA) is an immunosuppressant drug that is widely used to prevent organ rejection after transplantation and to treat numerous autoimmune disorders^1^. However, its clinical use is hampered by its nephrotoxicity and cardiovascular side effects (2, 3). In experimental studies, perimyocytic fibrosis has been reported in cyclosporine-treated male rats after heterotopic transplantation^2^. In addition, CSA negatively impacts cardiac contractility in the male rat isolated heart preparation^3^. Chronic CSA administration is accompanied by signs of cardiac ischemia, such as QTc prolongation and increases in T wave amplitude in male rats^4^. Clinically, CSA was shown to increase the oxidative capacities of cardiac tissue through the inhibition of complexes I and IV in the mitochondrial respiratory chain^5^.

Several mechanisms have been implicated in CSA toxicity. Studies from our laboratory suggested redox imbalances, e.g. reduced superoxide dismutase and elevated toxic aldehydes, played a key role in peripheral and central sites in the hypertension and cardiac autonomic defects caused by CSA^6^. The reversal of these effects by the superoxide dismutase mimetic tempol establishes a causal relationship between the oxidative insults and CSA effects (6). The upregulation of vascular endothelin ETA receptors also incite the CSA-induced hypertension and vascular dysfunctions^7^. Other cellular oxidative and inflammatory pathways were found to mediate the CSA-induced nephrotoxicity^8^. Nuclear factor kappa B (NFκB) is one of the most important key genes in controlling cellular inflammation and redox production^9^ and one of the most important mediators of CSA effects^10^. The activation of the renin-angiotensin system (RAS) and angiotensin AT_1_ receptors is predisposed to cardiac cellular oxidative stress generation^11^ and the blockade of these receptors protects against CSA nephrotoxicity^12^. On the other hand, adipokines such as adiponectin inhibit cellular inflammation and oxidative stress^13^. Furthermore, evidence suggests that the increase in vascular adiponectin might account for the counteraction by pioglitazone of hypertension and endothelial dysfunction caused by CSA^14^.

As the majority of the reported clinical^5^ and experimental^2^,^3^,^4^ studies which evaluated CSA cardiotoxicity were conducted on male subjects, it remains unclear as to whether this effect of CSA is sexually specific. This issue was addressed in the current study by determining the effects of chronic CSA administration on LV, ECG, and cardiac histopathological profiles in age-matched male and female rats. The roles of cardiac proinflammatory (NFκB and Angiotensin AT_1_ receptors) and antiinflammatory (adiponectin) cytokines in the developed CSA cardiotoxicity were also investigated.

## Results

### Effects of CSA on cardiac hemodynamics

**Figure 1:**
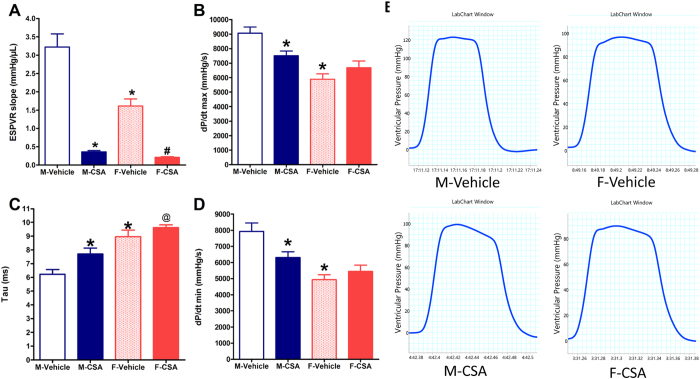
Effect of chronic administration of cyclosporine A (CSA, 15 mg/kg/day for 3 weeks, s.c.) on the end-systolic pressure volume relationship (ESPVR slope, **A**), the rate of rise in LV pressure (dP/dt_max_, **B**), the isovolumic relaxation constant (*Tau,* **C**) and the rate of fall in LV pressure (dP/dt_min_, **D**) in male (M) and female (F) rats. Data are presented as mean ± standard error of 6–8 animals in each group. *P < 0.05, compared with the corresponding M-Vehicle group values; ^#^P < 0.05, compared with the corresponding F–Vehicle group values; ^@^P < 0.05, compared with the corresponding M-CSA group values; by One Way ANOVA and Newman Keuls *post hoc* test. Panel E shows representative original recordings of ventricular pressure.

As shown in Fig. 1A,C, data obtained from control (vehicle-treated rats) groups showed that the indices of ventricular systolic (ESPVR slope) and diastolic (relaxation index, *Tau*) functions were significantly lower and higher, respectively, than respective values in age-matched male rats. The rates of rise (dP/dt_max_) and fall (dP/dt_min_) in LV pressure as well as BP values (SBP and DBP) were all significantly lower than corresponding male values (Fig. 1B,D and Table 1).

The subcutaneous administration of CSA (15 mg/kg/day sc) for 3 weeks resulted in a sex-dependent impairment in ventricular systolic and diastolic functions. The impaired systole was reflected by a 9.5 fold decrease in ESPVR slope in males and a 7.7 fold decrease in females (Fig. 1A) compared with the corresponding control. The impaired diastole was reflected by a 23% increase in *Tau* in males and a 16% increase in females (Fig. 1B) compared with the corresponding control. In line with this, CSA administration significantly decreased the rate at which LV pressure rose (dP/dt_max_) and fell (dP/dt_min_) in male rats but had no effect in female rats (Fig. 1B,D). The heart rate did not change significantly following CSA administration in male nor female rats, although female control rats had lower heart rate values (Table 1) compared with males. On the other hand, CSA had no effect on HR in either of the two rat sexes and caused significant increases in systolic and diastolic BP in female rats only (Table 1).

### Cardiac electrophysiological effects of CSA

**Figure 2:**
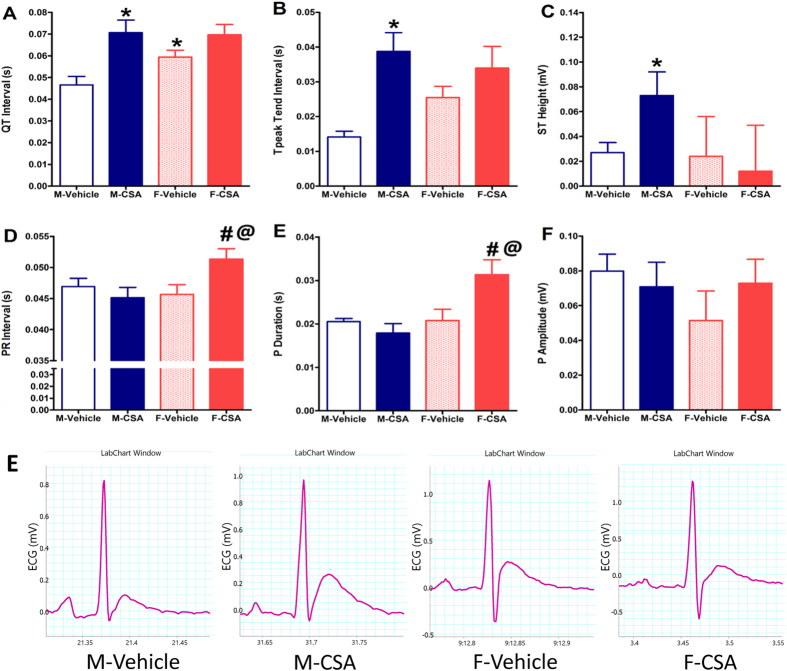
Effect of chronic administration of cyclosporine A (CSA, 15 mg/kg/day for 3 weeks, s.c.) on electrocardiograph QT (**A**), T peak trend (**B**), ST height (**C**), PR intervals (**D**), P duration (**E**) and P amplitude (**F**) in male (M) and female (F) rats. Data are presented as mean ± standard error of 6–8 animals in each group. P < 0.05, compared with the corresponding M-Vehicle group values; ^#^P < 0.05, compared with the corresponding F-Vehicle group values; ^@^P < 0.05, compared with the corresponding M-CSA group values; by One Way ANOVA and Newman Keuls *post hoc* test. Panel E shows representative original recordings of ECG.

With the exception of a significantly longer QT interval in vehicle-treated female rats compared with their male counterparts, the ECG characteristics were similar in both rat sexes (Fig. 2). More specifically, CSA caused significant increases in QT and T peak trend intervals and elevations in ST segment in male rats, but not in females (Fig. 2A–C), indicating LV ischemia and arrhythmogenesis. On the other hand, CSA resulted in a delay in AV conduction in female rats only, as reflected by the significant prolongation in P duration and PR interval and increase in P-amplitude (Fig. 2D–F).

### Effect of CSA on cardiac inflammatory and lipid profiles

**Figure 3:**
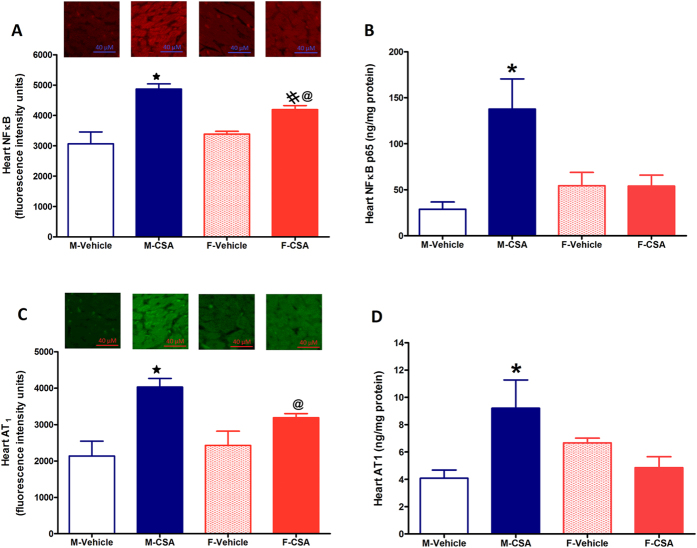
Effect of chronic administration of cyclosporine A (CSA, 15 mg/kg/day for 3 weeks, s.c.) on cardiac nuclear factor kappa B immunofluorescence and ELISA (NFκB, panel A,B) and angiotensin receptor type 1 immunofluorescence and ELISA (AT_1_, panel C,D) in male (M) and female (F) rats. Data are presented as mean ± standard error of 6–8 animals in each group. P < 0.05, compared with the corresponding M-Vehicle group values; ^#^P < 0.05, compared with the corresponding F-Vehicle group values; ^@^P < 0.05, compared with the corresponding M-CSA group values; by One Way ANOVA and Newman Keuls *post hoc* test. Micrographs at the top are representative fluorescence images of heart cross sections immunofluorescence stained by NFκB or AT_1_ antibodies followed by Alexa fluor conjugated secondary antibodies.

**Figure 4:**
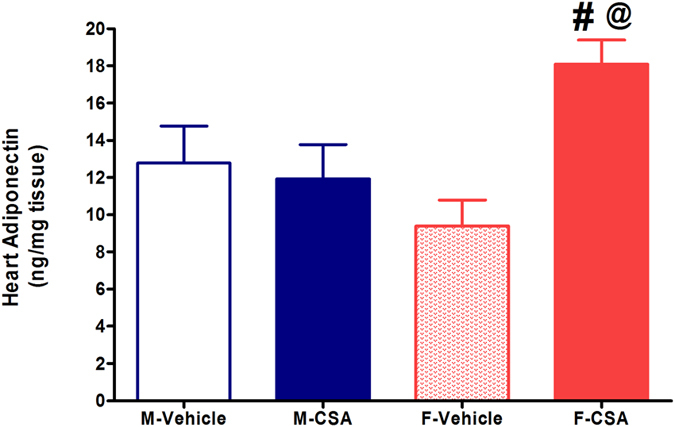
Effect of chronic administration of cyclosporine A (CSA, 15 mg/kg/day for 3 weeks, s.c.) on heart tissue levels of adiponectin in male (M) and female (F) rats. Data are presented as mean ± standard error of 6–8 animals in each group. P < 0.05, compared with the corresponding M-Vehicle group values; ^#^P < 0.05, compared with the corresponding F-Vehicle group values; ^@^P < 0.05, compared with the corresponding M-CSA group values; by One Way ANOVA and Newman Keuls *post hoc* test.

As shown in Fig. 3, immunofluorescence and ELISA studies showed similar levels of NFκB and AT_1_ receptors in the cardiac tissues of control male and female rats. Compared with vehicle-treated values, CSA significantly increased the cardiac levels of the inflammatory proteins NFκB and AT_1_ receptors in cardiac tissues of male rats by 70% and 100%, respectively. This is in stark contrast to the effect of CSA in female rats where it elicited elevations of around 20% in cardiac NFκB but had no effect on AT_1_ receptors (Fig. 3). The cardiac adiponectin level was significantly increased by CSA in female rats while it had no effect in male rats (Fig. 4). Serum cholesterol was elevated after CSA administration in male rats only (Table 2). Serum levels of uric acid or triglycerides were not affected by CSA in male or female rats.

### Cardiac histopathological effects of CSA

**Figure 5:**
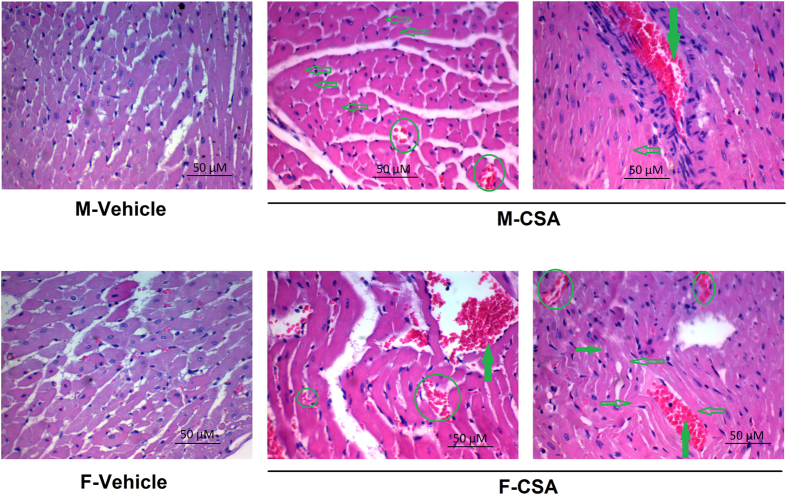
Photomicrographs (1200×, H & E) of heart sections obtained from male (M) and female (F) rats treated chronically with cyclosporine A (CSA, 15 mg/kg/day for 3 weeks, s.c.) or its vehicle. Hollow arrows point to cardiac muscle shrinkage and pyknosis. Solid arrows point to congested vascular space while circles point to extravascular blood cells between cardiac muscle.

CSA administration was associated with vascular congestion, blood extravasation, and a marked degeneration of cardiac muscle as indicated by the shrinkage of cardiac muscles and absence or pyknosis of cardiac muscle nuclei. These effects of CSA were demonstrated in both male and female rats (Fig. 5).

## Discussion

The present study is the first to investigate whether the sex of rats influences cardiotoxic manifestations caused by chronic administration of CSA. To perform this study, a multidisciplinary approach was employed that encompassed LV, electrocardiographic, histopathological, and inflammatory elements. The data generated clearly showed that male rats are more susceptible to the negative cardiac effects of CSA. The treatment of both rat sexes with CSA caused cardiac structural damage and LV dysfunction as suggested by the decreases and increases in ESPVR and Tau, respectively. The latter effects, however, were more noticeable in males. Moreover, CSA effects that appeared in male rats only were as follows: (i) ST elevations and increased QTc and T peak trend intervals on electrocardiograms, (ii) increases in blood cholesterol and LV NFκB and AT_1_ expressions, and (iii) reductions in the rates of rise (dp/dt_max_) and fall (dp/dt_min_) in LV pressure. In contrast, CSA delayed AV conduction and increased LV adiponectin in female rats. Together, compared with their female counterparts, CSA-treated male rats exhibited more deteriorated LV function and increased arrhythmogenesis and ischemic potential, due perhaps to exacerbated inflammatory conditions.

Recent evidence suggests that CSA negatively influences cardiac contractility and impairs systolic and diastolic functions via the modulation of Ca^2+^ currents in swine cardiomyocytes^15^. Similar observations of impaired cardiac contractility by CSA were demonstrated in rats in earlier studies^3^. This view is supported by the data in the present study, which employed ultra-miniature Millar catheter technology for measuring LV function. The data demonstrates the inhibitory effects of CSA on indices of cardiac dynamics such as ESPVR and Tau in both male and female rats. Notably, whereas ESPVR describes the maximum pressure that is generated by the ventricle at any given LV volume (end-systole), Tau represents the exponential decay of ventricular pressure during isovolumic relaxation and its prolongation might indicate diastolic dysfunction^16^,^17^. The discovery that these CSA effects were more pronounced in male than in female rats provides the first evidence that the cardiac effects of CSA might be sexually differentiated.

In fact, the notion that the male gender is more responsive to CSA cardiotoxicity is substantiated by several other findings in the present study. One advantage of the Millar pressure volume catheter is that it permits the measurement of the rate of rise (dP/dt_max_) and fall (dP/dt_min_) in LV pressure, global indices of LV contractility and relaxation^18^,^19^, respectively, independent of loading conditions and heart rate^16^. Here, we report that both functions (dP/dt_max_ and dP/dt_min_) were significantly reduced by CSA in male rats but had no effect in female rats. The reduced dp/dt_min_ is consistent with the delayed lusitropism (increased Tau) caused by CSA in the male rat, which infers an impaired diastolic function. Considering dP/dt is a powerful tool for assessing left ventricular function, fluctuations in the rate of rise or decline in LV pressure is believed to have prognostic implications in cardiac disorders such as heart failure^20^,^21^.

The electrocardiographic profile is one of the primary diagnostic tools for coronary ischemia and abnormal cardiac rhythms. Previous reports showed that CSA administration over 7 weeks produced signs of cardiac ischemia such as QTc prolongation and increases in T wave amplitude^4^. Nevertheless, the ischemic and arrhythmogenic effects of CSA vary widely and depend on the dose employed and animal species to which it is administered^22^. The ECG recordings collected from the current study provide several observations that reinforce the male specificity of the cardiotoxic effect of CSA. Most importantly, the significant elevations in ST segment and increases in QT and T peak trend intervals observed in CSA-treated male rats are consistent with increased propensity to LV ischemia, delayed ventricular repolarization, and arrhythmogenesis. Although these effects were not observed in CSA-treated female rats, signs of delayed AV conduction such as prolonged P wave duration and PR intervals were evident. This, however, did not appear to have impacted the cardiac dynamics considering the lack of any concomitant changes in heart rate in the female population.

Since the inflammatory response is pivotal in the initiation and progression of arrhythmogenic cardiomyopathy^23^,^24^, we tested the possibility that imbalances in the proinflammatory/antiinflammatory settings might explain the preferential vulnerability of male rats to the cardiotoxic effect of CSA. Along this line, immunofluorescence and ELISA measurements of the present study revealed the novel finding that CSA led to the upregulation of the cardiac expression of the inflammatory angiotensin AT_1_ receptors in male but not female rats. Renin angiotensin system abnormalities are considered important risk factors for cyclosporin nephrotoxicity in patients with psoriasis^25^. An incremental effect of CSA on the density of AT_1_ receptors has been reported in mouse medullary thick ascending limb cells^26^. In addition, the present study showed that CSA resulted in significantly greater increases in cardiac expression of the cardiac inflammatory mediators NFκB in male compared with female rats. Evidence from other reports indicates that CSA augments the growth of A431 epidermoid carcinoma xenograft tumors by activating NFκB signaling^27^. Furthermore, elevation in the adiponectin level in cardiac tissues in the present work was observed in female rats only. Notably, adiponectin is a cytokine that combats cellular inflammation and oxidative stress^13^ and mediates the favorable effect of pioglitazone against CSA vasculopathy^14^. Moreover, transgenic mice with adiponectin overexpression are tolerant to the aldosterone-induced left ventricular hypertrophy and diastolic dysfunction^28^. Together, the less evident inflammatory response along with the increased cardiac adiponectin may account for the diminution of CSA cardiotoxicity in female rats. In a similar way to the present study, Diwan *et al*.^29^ showed that the inflammatory kidney damage induced by adenine was more enhanced in male than in female rats due to the suppression of renal estrogen receptor α expression, which reinforces the importance of estrogen signaling in alleviating inflammatory insults.

Considering the paradoxical roles for estrogen (protection) and testosterone (exacerbation) in cardiovascular pathologies^30^, it is imperative to comment on the possible interrelationship between gonadal sex hormones and RAS in the sex-dependent CSA cardiotoxicity. Among several other factors, the molecular basis of the sex specificity in the incidence of cardiovascular disease relates to RAS activity, which is inhibited by estrogen and stimulated by testosterone^31^. Moreover, ventricular ACE expression is more abundant in male than in female mice at both mRNA and protein levels, and gonadectomy elicits reciprocal changes in ACE expression in the ventricular tissues of females (increases) and males (decreases)^32^. Increased ACE abundance in hypertrophied and failing hearts contributes to the local generation of angiotensin II and consequent cardiac remodeling^33^,^34^,^35^ and implicates androgen in the increased incidence of angiotensin II-induced aortic aneurysms in male mice as well as in testosterone-treated female mice. It is tempting to speculate that the directionally opposite effects of sex hormones or their downstream effectors on cardiac inflammatory and RAS profiles might underlie the sex differences in CSA cardiotoxicity. However, as age-matched male and female rats of 6-weeks old were used in the current study, whether similar sex-related patterns of CSA cardiotoxicity would be seen in younger or older rats remains to be seen. Thus, further studies are clearly needed to investigate this possibility.

### Study limitations

While we report here on the interaction of a 3-week CSA regimen with cardiac performance and its sex dependence in rats, different features may develop with longer-term (months or years) regimens. In addition, because the female studies were performed in randomly cycling rats, it remains unclear as to whether cyclic fluctuations in sex hormonal levels could have impacted the data. The translational information collected from the current study also needs to be corroborated clinically.

## Conclusion

In summary, the current study establishes the importance of rat sex in defining the magnitude of CSA cardiotoxicity. While functional and histopathological signs of LV damage were evident in both sexes, electrocardiographic and Millar electrophysiological studies revealed more deterioration in LV systolic and diastolic functions in the male gender together with arrhythmogenic and ischemic signs due perhaps to the concomitant deterioration of the inflammatory state.

## Methods

### Animals

This study was performed in accordance with the Kingdom of Saudi Arabia Research Bioethics and Regulations. Male and female Wistar rats (6 weeks old; King Abdulaziz University, Kingdom of Saudi Arabia) were housed (3–4 rats per cage) in clear polypropylene cages and maintained under constant environmental conditions with equal light–dark cycles. Rats had free access to a commercially available rodent pellet diet and water.

### Study design and protocols

The experimental protocol was approved by the Unit of Biomedical Ethics Research Committee, King Abdulaziz University, Kingdom of Saudi Arabia. Animals were randomly divided into four groups (8 animals each) of control males (M-Vehicle), cyclosporine-treated males (M-CSA), control females (F-Vehicle) and cyclosporine treated females (F-CSA). CSA was injected subcutaneously at a dose of 15 mg/kg/day^14^,^36^ for 21 consecutive days, while control rats received the vehicle (18% koliphore, 2% ethanol in sterile saline). At the end of the study, control animals were anesthetized by the intraperitoneal injection of ketamine (100 mg/kg)/xylazine (10 mg/kg) while CSA-treated animals received a smaller dose of ketamine/xylazine (50/8 mg/kg) as the normal dose was found to be lethal to these rats^37^. Then, cardiac hemodynamics and blood pressure were recorded using a microtip catheter inserted in the right carotid artery through a small opening in the artery and advanced to the left ventricle as described in our previous studies^38^. Cardiac conductivity was determined by surface ECG^39^. After a 15-min recording of basal cardiac contractility and conductivity, 4 ml of blood was withdrawn from the vena cava (through a small incision in the lower abdomen), was allowed to coagulate for 30 min at 4 °C, and was then centrifuged (3000 × g, 4 °C, 20 min). The serum was aspitated and divided into aliquots and stored at −80 °C for later analysis of uric acid, triglycerides and total cholesterol. The heart was quickly excised and a piece of the left ventricle (~50 mg) was snap frozen and stored at −80 °C for later analysis of adiponectin and protein content. The remaining part of the heart was fixed in 10% neutral buffered formalin for immunofluorescence detection of NFκB and angiotensin AT_1_ receptor expression and histopathological examination (hematoxylin and eosin stain).

### Hemodynamic recording

Invasive real time recording of cardiac hemodynamic was performed according to the method described in our previous reports^38^,^40^. Following anesthetisation as described above, the animals’ body temperature was maintained at 37 °C using a rectal probe and controlled heating pads. A microtip pressure volume catheter (PV catheter, SPR-901, Millar Instruments, Houston, TX, USA) was inserted through a small incision into the right carotid artery and advanced into the left ventricle. The PV catheter is capable of measuring both ventricular pressure and volume simultaneously and continuously from the intact beating hearts of rats. In addition, the other pressure sensor records arterial pressure at the mean time. After a 5 min stabilization period, the signals were continuously recorded. The microtip catheter was connected to a Power Lab Data Interface connected to a PC running Lab Chart professional software (v8.0, AD Instruments, Bella Vista, Australia) including the PV and blood pressure (BP) modules. The PV module analyzes the relationship between the LV pressure and volume signals and calculates the end-systolic pressure volume relationship (ESPVR) slope, isovolumic relaxation constant (*Tau*), rate of rise in LV pressure (dP/dt_max_), rate of fall in LV pressure (dP/dt_min_), developed Pressure (Pdev) and heart rate (HR). Systolic and diastolic BP was monitored via the BP module.

### Electrocardiogram (ECG) recording

The standard surface ECG was recorded according to the method previously described in a recent publication from our laboratory^40^ using a Powerlab^®^ system (AD Instruments, Bella Vista, Australia) connected to a PC running LabChart professional software with the ECG module. The ECG module quantitatively determines different components of the ECG.

### Biochemical analysis

Serum levels of uric acid, triglycerides, and total cholesterol were determined using the ELITech^®^ assay kit (ELITech, Puteaux, France). Heart adiponectin, AT1R and NFκB p65 were measured by ELISA using antibodies raised against rat adiponectin (Abcam, Cambridge, MA, USA), AT1R (LifeSpan Biosceinces, WA, USA), and NFκB p65 (Abcam, Bristol, UK), respectively.

### Immunofluorescence Studies

Immunofluorescence staining of NFκB and AT_1_ in rat paraffin embedded heart sections (5 μm) was carried out according to the method used in our previous work^41^,^42^,^43^. Fixed heart tissue section slides were deparaffinized in xylene and rehydrated in ethanol and distilled water. Then, perforation was carried out by incubation with methanol at −20 °C for 30 min followed by washing with distilled water. Epitopes were retrieved (antigen retrieval) in a citrate buffer for 30 min at 95 °C and were then washed with phosphate buffered saline (PBS). Slides were then immediately transferred into a humidity chamber. Nonspecific binding sites were blocked (bovine serum albumin in PBS containing 5% normal goat serum, 1% bovine serum albumin, 0.1% Triton) at room temperature for 1 h. After the blocking, sections were washed (3 5 min) with PBS. Heart sections were then incubated with the intended primary antibody diluted in blocking buffer at 4 °C overnight. The sections were then washed (3 × 5 min) with PBS followed by incubation with a fluorescent conjugated secondary antibody (diluted 1:200 in blocking buffer) for 1 h in the dark. Sections were then washed (3 × 5 min) with PBS and slides were dried and mounted with ‘ProLong’ mounting media (Life Technologies, Paisley, UK). The slides were stored in the dark overnight before examination with a Zeiss AXIO OBSERVER D1 fluorescent microscope (Carl Zeiss, Gottingen, Germany) using two filters at 470/40 and 545/25 nm excitation, 495 and 570 nm beam splitter, and 525/50 and 605/70 nm emission respectively. Images were acquired with identical acquisition parameters, with minimum excitation and gain. Quantitative comparisons of image fluorescence were made with ZEN software (Carl Zeiss, Gottingen, Germany). For presentation purposes, the level of the fluorescence images were equally adjusted after the fluorescence quantifications were carried out on non-manipulated images. Sections treated with the secondary antibody alone did not show specific fluorescence while incubating the primary antibody with the blocking peptide significantly reduced the signal. The primary antibodies used were: rabbit polyclonal anti NFκB (1:1000, Abcam, Cambridge, MA, USA), mouse monoclonal anti AT_1_ receptor (1:133, Abcam, Cambridge, MA, USA). The secondary antibodies used were Alexa Fluor (λex = 488) conjugated goat anti-mouse and Alexa Fluor (λex = 594) conjugated goat anti-rabbit (1:200, Life Technologies, Grand Island, NY, USA).

### Drugs and chemicals

The following drugs and chemicals were used: cyclosporine (Sandimmune^®^, Novartis Pharmaceuticals Corporation East Hanover, New Jersey), ketamine (Tekam^®^, Hikma Pharmaceutical, Amman, Jordan), xylazine (Seton^®^, Laboratories Calier, Barcelona, Spain) (Sigma-Aldrich, St. Louis, MO, USA).

### Statistical analysis

Values are expressed as mean ± SEM. Statistical analysis was carried out by analysis of variance (ANOVA) followed by Newman-Keuls’ post hoc test using statistical software (Prism 5, GraphPad, CA, USA). Probability levels less than 0.05 were considered statistically significant.

## Additional Information

**How to cite this article**: El-Bassossy, H. M. *et al*. The inflammatory state provokes sexual dimorphism in left ventricular and electrocardiographic effects of chronic cyclosporine in rats. *Sci. Rep.* **7**, 42457; doi: 10.1038/srep42457 (2017).

**Publisher's note:** Springer Nature remains neutral with regard to jurisdictional claims in published maps and institutional affiliations.

## Figures and Tables

**Table 1:** Effect of daily administration of cyclosporine A (CSA, 15 mg/kg, sc) on the heart rate, systolic blood pressure (SBP) and diastolic blood pressure (DBP) in male (M) and female (F) rats.

Treatment	Heart rate (BPM)	SBP (mmHg)	DBP (mmHg)
M-Vehicle	321.0 ± 19.4	107 ± 4.5	81.8 ± 4.2
M-CSA	312.8 ± 8.5	118 ± 3.9	87.4 ± 3.4
F-Vehicle	261.7 ± 18.8*	88.9 ± 3.3*	64.3 ± 3.4*
F-CSA	269.3 ± 8.2^@^	107.1 ± 4.7^#^	83.5 ± 3.9^#^

Values are expressed as the mean ± S.E of mean; N = 6–8 animals; *P < 0.05, compared with the corresponding M-Vehicle group values; ^#^P < 0.05, compared with the corresponding F-Vehicle group values; ^@^P < 0.05, compared with the corresponding M-CSA group values; by One Way ANOVA and Newman Keuls *post hoc* test.

**Table 2:** Effect of daily administration of cyclosporine A (CSA, 15 mg/kg, sc) on the blood level of uric acid, triglycerides and total cholesterol in male (M) and female (F) rats.

Treatment	Uric acid (mg/dl)	Triglycerides (mg/dl)	Total cholesterol (mg/dl)
M-Vehicle	6.4 ± 0.2	63.5 ± 8.2	91.8 ± 7.2
M-CSA	5.7 ± 0.3	54.2 ± 13.3	142.7 ± 12.7*
F-Vehicle	5.5 ± 0.8	57.7 ± 4.7	112.0 ± 5.3
F-CSA	5.7 ± 0.5	59.1 ± 8.0	87.3 ± 7.4^@^

Values are expressed as the mean ± S.E of mean; N = 6–8 animals; *P < 0.05, compared with the corresponding M-Vehicle group values; ^@^P < 0.05, compared with the corresponding M-CSA group values; by One Way ANOVA and Newman Keuls *post hoc* test.
